# Initial Dosage Recommendation for Sirolimus in Children With Tuberous Sclerosis Complex

**DOI:** 10.3389/fphar.2020.00890

**Published:** 2020-06-11

**Authors:** Dong-Dong Wang, Xiao Chen, Hong Xu, Zhi-Ping Li

**Affiliations:** ^1^Department of Pharmacy, Children's Hospital of Fudan University, Shanghai, China; ^2^Department of Nephrology, Children's Hospital of Fudan University, Shanghai, China

**Keywords:** initial dosage, recommendation, sirolimus, children, tuberous sclerosis complex

## Abstract

Sirolimus is already used in the treatment of tuberous sclerosis complex (TSC), however, with narrow therapeutic range and considerable inter- and intra-individual pharmacokinetic variability, making it hard to develop an appropriate sirolimus initial dosage regimen, especially in children with TSC. The aim of this study was to recommend the optimal sirolimus initial dosing regimen in pediatric patients with TSC. Underlying physiological and genetic factors were collected to explore the effects on clinical sirolimus concentrations by establishing a nonlinear mixed effect (NONMEM) model, and to further simulate the optimal sirolimus initial dosing regimen using Monte Carlo method in pediatric patients with TSC. The once-daily regimen and the twice-daily regimen were recommended, respectively. For once-daily regimen, the dosages of 0.10, 0.07, 0.05, 0.04, 0.03 mg/kg/day were recommended for children with weights of 5–10, 10–20, 20–30, 30–50, and 50–60 kg, respectively. For twice-daily regimen, the dosages of 0.04, 0.03, 0.02 mg/kg/day (the daily dose was divided evenly into two doses) were recommended for children with weights of 5–20, 20–40, 40–60 kg, respectively. The initial dosages of sirolimus in children with TSC were recommended for the first time.

## Introduction

Tuberous sclerosis complex (TSC), a genetic autosomal dominant disorder, is caused by constitutive activation of mammalian target of rapamycin complex 1 (mTORC1) due to mutations in genes coding for hamartin (*TSC1*) or tuberin (*TSC2*) proteins, and its incidence is 0.10–0.17‰ (Osborne et al., 1991; Crino et al., 2006; Curatolo et al., 2008). The *TSC1* or *TSC2* genes pathogenic changes lead to upregulation of the mechanistic target of rapamycin (mTOR) signal pathway, in charge of synaptic plasticity, protein synthesis, cell growth, proliferation, differentiation, and migration, which has relationship with the formation of benign hamartomas in the brain, heart, liver, kidneys, lungs, skin and retina etc. (van der Poest Clement et al., 2020). Therefore, patients with TSC have constitutive activation of mTOR resulting in hamartomas in the above tissues (Ebrahimi-Fakhari et al., 2018).

Sirolimus, an mTOR inhibitor, has shown good effects on multiple manifestations of TSC, which has been approved for treating TSC (Franz and Capal, 2017). The biggest difference from other treatments for TSC is that sirolimus works directly on the potential pathogenesis of TSC, not its symptoms (Ebrahimi-Fakhari et al., 2019). However, with narrow therapeutic range and considerable inter- and intra-individual pharmacokinetic variabilities, making it hard to develop a sirolimus initial dosage regimen, especially in children with TSC. The aim of this study was to explore the effects of underlying physiological and genetic factors on clinical sirolimus concentrations by establishing a nonlinear mixed effect (NONMEM) model, and to further simulate the optimal sirolimus initial dosing regimen using Monte Carlo method in pediatric patients with TSC.

## Methods

### Patients

Pediatric patients from June 2016 to September 2019 at the Children's Hospital of Fudan University (Shanghai, China) were collected, retrospectively. Partial basic clinical dataset of some children were collected from a previous research (Wang et al., 2018). The criteria for inclusion were as follows: (i) aged <16 years old, (ii) diagnosed with TSC, (iii) treated by sirolimus, (iv) therapeutic drug monitoring (TDM) for sirolimus, (v) blood samples can be obtained for pharmacogenomics analysis. Exclusion criteria: patients with medications which effect sirolimus metabolism, mainly including CYP 3A4 inhibitors or inducers, and P-gp inhibitors or inducers. The study was approved by the Research Ethics Committee of Children's Hospital of Fudan University (Ethical code: [2019] 019). As for the study was retrospective and blood samples for pharmacogenomics analysis were leftover or discarded specimens from TDM, the analysis was approved by the ethics committee of our hospital without the need for written informed consent.

### TDM and Pharmacogenomic Analysis

Sirolimus concentrations were tested with the Emit 2000 Sirolimus Assay (Siemens Healthcare Diagnostics Inc.) with range of linear response, 3.5–30 ng/ml, whose values of inter-assay variability [coefficient of variation (CV%)] <4.0%, and values of intra-assay CV (%) <6.2%.

The blood samples used for pharmacogenomic testing came from TDM residual samples, and the analysis was measured by Admera Health (Suzhou, China) with PGxOne^®^160 *via* the Illumina X10 Sequencing System. Hardy–Weinberg equilibrium was investigated with STATA computer software (version 12.0, Stata Corp LP, USA) and the value of *P <*0.05 was considered significant from a statistical point of view.

### Population Pharmacokinetic Model

Dataset were used to build population pharmacokinetic model using the non-linear mixed-effects modeling software, NONMEM (edition 7, ICON Development Solutions, Ellicott City, MD, USA) and a first-order conditional estimation method with interaction (FOCE-I method). The pharmacokinetic parameters included apparent oral clearance (CL/F), volume of distribution (V/F), and absorption rate constant (Ka), whose value was fixed at 0.485/h (Wang et al., 2018; Wang et al., 2019a).

### Random Effect Model

Equation (1) showed the inter-individual variability:

(1) $${\text{P}}_{\text{i}}=\text{TV}(\text{P})\times \text{exp}\;({\text{η}}_{i})$$

P_i_ was on behalf of the individual parameter value and TV(P) represented the typical individual parameter value. η_i_ was symmetrical distribution, which was random term with zero mean and variance omega^2^ (ω^2^).

Equation (2) showed the random residual variability:

(2) $$\text{OB}{\text{S}}_{\text{i}}=\text{PR}{\text{E}}_{\text{i}}\times (1+\epsilon_{1})+\epsilon_{2}$$

OBS_i_ was the observed concentration, PRE_i_ was the individual predicted concentration and ϵ_1_ and ϵ_2_ were symmetrical distribution, which was random term with zero mean and variance sigma^2^ (σ^2^).

### Covariate Model

Equation (3) showed the relation of pharmacokinetic parameters with weight:

(3) $${\text{P}}_{\text{i}}={\text{P}}_{\text{std}}\times {\left({\text{W}}_{i}/{\text{W}}_{\text{std}}\right)}^{\text{index}}$$

P_i_ represented the i-th individual parameter, W_i_ represented the i-th individual weight. W_std_ was the standard weight of 70 kg. P_std_ was the typical individual parameter, whose weight was W_std_. index was the allometric coefficient: 0.75 for the CL/F and 1 for the V/F (Anderson and Holford, 2008).

Equations (4) showed pharmacokinetic parameters and genotype:

(4) $${\text{P}}_{\text{i}}=\text{TV}(\text{P})\times \theta^{\text{genotype}}$$

Equations (5) and (6) showed pharmacokinetic parameters and the other continuous covariates or categorical covariates, respectively:

(5) $${\text{P}}_{\text{i}}=\text{TV}(\text{P})\times {\left({\text{Cov}}_{\text{i}}/{\text{Cov}}_{\text{median}}\right)}^{\theta }$$

(6) $${\text{P}}_{\text{i}}=\text{TV}(\text{P})\times (1+\theta \times {\text{Cov}}_{\text{i}})$$

P_i_ was the individual parameter value, TV(P) was the typical individual parameter value. θ was the parameter to be estimated and Cov_i_ was the covariate of the i-th individual. Cov_median_ was the population median for the covariate.

Changes of objective function value (OFV) was calculated using covariate inclusions and a decrease in the OFV >3.84 (*P <* 0.05, degree of freedom = 1) was used as a criterion for inclusion of the covariate in the base model. When a full regression model was built, the model was further testified by dropping the covariate from each parameter one at a time to acquire the final model. An increase in the OFV >6.64 (*P <* 0.01, degree of freedom = 1) was used as a criterion for retaining significant covariate–parameter relationships in the model (Wang et al., 2020).

### Model Evaluation

The final model was estimated by individual plots, distribution of weighted residuals for model (density *vs.* weighted residuals, and quantiles of weighted residuals *vs.* quantiles of normal), goodness-of-fit plots of model (observations *vs.* population predictions, observations *vs.* individual predictions, absolute value of weighted residuals of individual (│iWRES│) *vs.* individual predictions, weighted residuals *vs.* time), and visual predictive check (VPC) of model. In addition, bootstrap method was used to repeated random sampling with replacement from the raw data base with 1,000 repetitions with different random sampling. The medians and 2.5th–97.5th percentiles of the results from bootstrap were used for comparing with final model parameters.

### Simulation

The influence of sirolimus initial doses on the probability to achieve the target concentration (5–15 ng/ml) (MacKeigan and Krueger, 2015; Nathan et al., 2015) were simulated by Monte Carlo method based on a once-daily regimen or a twice-daily regimen. In every case, 1,000 virtual patients were simulated in each of the seven weight groups (5, 10, 20, 30, 40, 50, and 60 kg) and for ten doses (0.01, 0.02, 0.03, 0.04, 0.05, 0.06, 0.07, 0.08, 0.09, and 0.10 mg/kg/day). The twice-daily regimen was split evenly into two doses a day.

## Results

### Patient Data

A total of 15 children with TSC, included seven boys and eight girls and whose ages were from 1.08 to 13.95 years old. The demographic data of patients was shown in **Table 1**. Partial basic clinical dataset of some children were collected from a previous research (Wang et al., 2018). **Table 2** showed pharmacogenetics analysis and Hardy–Weinberg equilibrium. The P value of Pearson's Chi-squared test from every gene >0.05, showing Hardy–Weinberg equilibrium.

**Table 1:** Demographic data of patients.

Characteristic	Mean ± SD	Median (range)
**Gender (boys/girls)**	7/8	/
**Age (years)**	6.16 ± 2.80	5.75 (1.08–13.95)
**Weight (kg)**	23.83 ± 8.88	22.00 (10.00–50.00)
**Albumin (g/L)**	45.59 ± 2.29	46.10 (40.30–48.30)
**Alanine transaminase (IU/L)**	8.18 ± 6.15	7.00 (1.00–28.00)
**Aspartate transaminase (IU/L)**	23.31 ± 6.38	24.00 (10.00–37.00)
**Creatinine (μmol/L)**	31.97 ± 10.41	29.00 (16.00–59.00)
**Urea (mmol/L)**	4.71 ± 1.27	4.50 (1.80–8.20)
**Total protein (g/L)**	71.81 ± 4.75	70.60 (64.70–81.10)
**Total bile acid (μmol/L)**	3.47 ± 2.51	2.60 (1.30–11.80)
**Direct bilirubin (μmol/L)**	2.01 ± 1.02	1.70 (0.40–4.80)
**Total bilibrubin (μmol/L)**	5.91 ± 3.65	5.00 (1.00–17.70)
**Hematocrit (%)**	37.86 ± 2.88	37.75 (30.80–45.30)
**Hemoglobin (g/L)**	126.44 ± 10.15	128.50 (107.00–147.00)
**Mean corpuscular hemoglobin (pg)**	27.59 ± 1.35	27.45 (25.10–31.00)
**Mean corpuscular hemoglobin concentration (g/L)**	334.15 ± 13.01	332.00 (309.00–364.00)

**Table 2:** Pharmacogenetics analysis and Hardy–Weinberg equilibrium.

Gene	Variation	Genotype	Frequency	%	P value ^a^
**ABCB1**	rs1045642	A/A	1	6.67	0.7913
		A/G	5	33.33	
		G/G	9	60.00	
**ABCC4**	rs1751034	C/C	1	6.67	0.1013
		C/T	2	13.33	
		T/T	12	80.00	
**ABCC8**	rs757110	A/A	3	20.00	0.7821
		A/C	8	53.33	
		C/C	4	26.67	
**ABCG2**	rs2231142	G/G	8	53.33	0.9299
		G/T	6	40.00	
		T/T	1	6.67	
**CYP2C9**		*1/*1	12	80.00	–
		*3/c.820-6326A>C	2	13.33	
		*1/c.820-6326A>C	1	6.67	
**CYP2C19**		*1/*1	5	33.33	–
		*1/*2	5	33.33	
		*2/*2	3	20.00	
		*1/*3	1	6.67	
		*1/*17	1	6.67	
**CYP3A4**		*1A/*1A	9	60.00	–
		*1A/*1G	6	40.00	
**CYP3A5**		*1/*3	7	46.67	–
		*3/*3	8	53.33	
**CYP4F2**		*1/*1	6	40.00	–
		*1/*3	8	53.33	
		*3/*3	1	6.67	
**UGT1A1**		*1/*1	9	60.00	–
		*1/*6	2	13.33	
		*6/*6	1	6.67	
		*28/*80	2	13.33	
		*28/*28/*80	1	6.67	
**UGT1A8**	rs1042597	C/C	3	20.00	0.1213
		C/G	4	26.67	
		G/G	8	53.33	
**UGT2B15**	rs1902023	A/A	7	46.67	0.6985
		A/C	6	40.00	
		C/C	2	13.33	

a Pearson chi-squared test.

### Modeling and Evaluation

The final population pharmacokinetics model was as follow:

(7) $$\text{CL}/\text{F}=6.48\times {\left(\text{weight}/70\right)}^{0.75}$$

(8) V/F=124×(weight/70)

**Figure 1:**
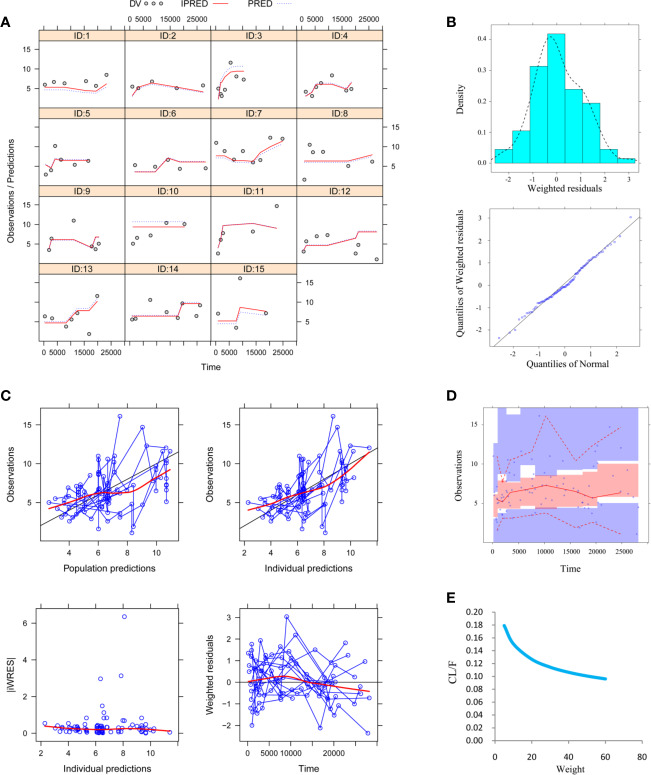
Model evaluation. **(A)** Individual plots. **(B)** Distribution of weighted residuals for model. Density *vs.* weighted residuals, and quantiles of weighted residuals *vs.* quantiles of normal. **(C)** Goodness-of-fit plots of model. Observations *vs.* population predictions, observations *vs.* individual predictions, absolute value of weighted residuals of individual (│iWRES│) *vs.* individual predictions, weighted residuals *vs.* time. **(D)** Visual predictive check (VPC) of model. The middle solid line represents the median of the prediction-corrected concentrations. The lower and upper dashed lines are the 2.5th and 97.5th percentiles of the prediction-corrected concentrations. **(E)** Sirolimus apparent clearance rate (CL/F, L/h/kg) of pediatric tuberous sclerosis complex patients with different weight. Partial concentration values were collected in a previous study (Wang et al., 2018).

**Figure 1A** was individual plots of all 15 pediatric patients with TSC, demonstrating acceptable predictability from the perspective of clinical sparse data. **Figure 1B** was distribution of weighted residuals for final model and the distribution was normal. **Figure 1C** was goodness-of-fit plots of final model. **Table 3** was parameter estimates of the final model and bootstrap validation. **Figure 1D** was VPC of final model and most of the observed sirolimus concentrations were within the 95% prediction intervals from the simulation data, indicating that the prediction-corrected concentrations were well predicted by the final model.

**Table 3:** Parameter estimates of final model and bootstrap validation.

Parameter	Estimate	SE (%)	Bootstrap		Bias (%)
			Median	95% Confidence interval	
**CL/F (L/h)**	6.48	33.2	6.65	[2.67, 13.60]	2.62
**V/F (L)**	124	69.8	133	[27, 2126]	7.26
**Ka (h^−1^)**	0.485 (fixed)	–	–	–	–
**ω_CL/F_**	0.066	70.9	0.050	[0.003, 0.159]	−24.24
**ω_V/F_**	0.003	60.4	0.003	[0.003, 0.178]	0
**σ_1_**	0.312	27.2	0.306	[0.003, 0.415]	−1.92
**σ_2_**	1.249	54.8	1.153	[0.010, 2.332]	−7.69

95% confidential interval was displayed as the 2.5th, 97.5th percentile of bootstrap estimates. CL/F, apparent oral clearance (L/h); V/F, apparent volume of distribution (L); Ka, absorption rate constant (h^−1^); ω_CL/F_, inter-individual variability of CL/F; ω_V/F_, inter-individual variability of V/F; σ_1_, residual variability, proportional error; σ_2_, residual variability, additive error; Bias, prediction error, Bias = (Median-Estimate)/Estimate×100%.

### Simulation

**Figure 2:**
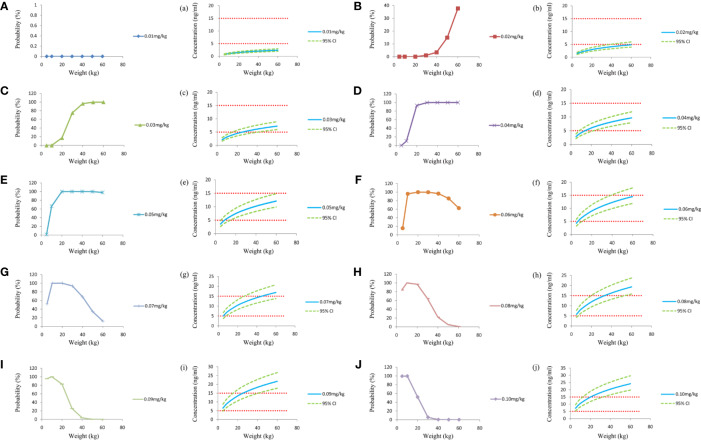
Simulation of sirolimus concentrations at different initial dosages once a day. **(A–J)** The probability to achieve the target concentration (5–15 ng/ml) at different initial dosages. a–j, sirolimus concentrations at different initial dosages. CI, confidence interval.

**Figure 3:**
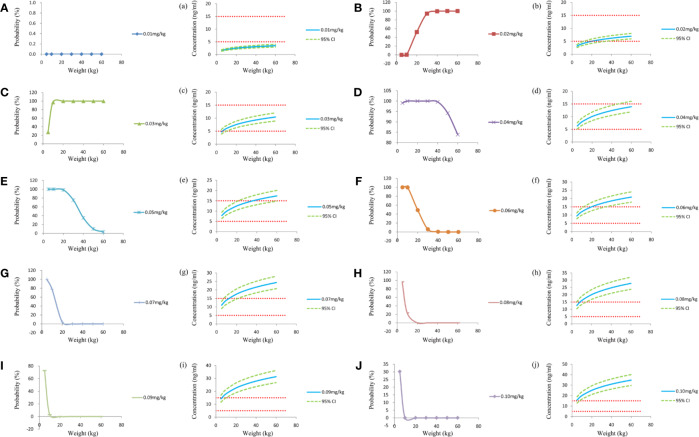
Simulation of sirolimus concentrations at different initial dosages which were split evenly into two doses a day. **(A–J)** The probability to achieve the target concentration (5–15 ng/ml) at different initial dosages. a–j, sirolimus concentrations at different initial dosages. CI, confidence interval.

**Figure 1E** was the clearance rate of sirolimus in children with TSC of different weights and we found that clearance rate range was about 0.18–0.10 L/h/kg from children with weight of 5–60 kg. According to the final model, we carried out the simulation. Based on a once-daily regimen or a twice-daily regimen, we simulated two sirolimus initial dosing regimens, as shown in **Figure 2** (a once-daily regimen) and **Figure 3** (a twice-daily regimen). The probability of reaching the target concentrations (5–15 ng/ml)(MacKeigan and Krueger, 2015; Nathan et al., 2015) and the 95% confidence interval of sirolimus concentrations were both considered, in other words, it was necessary to consider both safety and effectiveness. Finally, as shown in **Table 4**, for once-daily regimen, the dosages of 0.10, 0.07, 0.05, 0.04, 0.03 mg/kg/day were recommended for children with weights of 5–10, 10–20, 20–30, 30–50, and 50–60 kg, respectively; for twice-daily regimen, the dosages of 0.04, 0.03, 0.02 mg/kg/day (the daily dose was divided evenly into two doses) were recommended for children with weights of 5–20, 20–40, 40–60 kg, respectively.

**Table 4:** Initial dose recommendation of sirolimus in pediatric tuberous sclerosis complex.

Once a day		Split evenly into two doses a day	
**Body weight (kg)**	Dosage (mg/kg/day)	Body weight (kg)	Dosage (mg/kg/day)
**5–10**	0.10	5–20	0.04
**10–20**	0.07	20–40	0.03
**20–30**	0.05	40–60	0.02
**30–50**	0.04		
**50–60**	0.03		

## Discussion

Sirolimus, also known as rapamycin, is a macrolide antibiotic immunosuppressant, which has been widely used for liver transplantation (Song et al., 2019; Zhang et al., 2019), kidney transplantation (Ferron et al., 1997; Jiao et al., 2009), kaposiform hemangioendothelioma (Wang et al., 2019a) etc. In addition, it was also been reported that sirolimus has been used for TSC (Franz and Capal, 2017). However, with narrow therapeutic range and considerable inter- and intra-individual pharmacokinetic variabilities, making it hard to develop a sirolimus initial dosage regimen, especially in children with TSC. In clinical practice, TDM was used to monitor sirolimus concentrations and further adjust the next dose according to the concentration results. However, the initial dose cannot be recommended by this method, traditional TDM.

Fortunately, the combination of population pharmacokinetics and Monte Carlo simulation can successfully predict and recommend the best initial dosing regimen, the method has been widely used in clinical practice (Niu et al., 2019; Ren et al., 2019; Tang et al., 2019; Wang et al., 2019b). For example, Ren et al. (2019) reported population pharmacokinetics of voriconazole and optimization of dosage regimens based on Monte Carlo simulation in patients with liver cirrhosis, Niu et al. (2019) reported that population pharmacokinetics and dosing regimen optimization of lopinavir in Chinese adults infected with HIV, Wang et al. (2019b) reported population pharmacokinetics and initial dosing regimen optimization of cyclosporin in pediatric hemophagocytic lymphohistiocytosis patients, Tang et al. (2019) reported population pharmacokinetics and dosing optimization of amoxicillin in neonates and young infants. Based on these studies, the present study was to establish a population pharmacokinetics model of sirolimus in pediatric patients with TSC, and to further simulate the optimal sirolimus initial dosing regimen using Monte Carlo method.

In our final model, body weight was included as a covariate in models that affected sirolimus clearance. As many studies have reported, there was a non-linear relationship between drug clearance and body weight in pediatric patients, and it may be well described with allometric scaling using a coefficient of 0.75 for clearance and 1 for volume (Anderson and Holford, 2008; Hao et al., 2018). It is well known that the polymorphism of *CYP3A4* and *CYP3A5* affect sirolimus metabolism among adults to some extent (Tamashiro et al., 2017; Zhang et al., 2017). However, interestingly, during our modeling process, the polymorphism of *CYP3A4* and *CYP3A5* were not successfully included as covariates based on our sirolimus concentrations in clinical practice from pediatric patients with TSC. The main reason may be that our population of children has a wide age span, and the gene expression in different age development process is developmentally dependent and the expression levels of CYP3A isoforms were highly variable after birth (Lacroix et al., 1997). In other words, the activity of drug metabolizing enzymes of the same CYP3A genotype may be different in different age groups and genotypes may not accurately explain the differences from sirolimus concentrations in present study. It's not independent, in Mizuno et al.'s (Mizuno et al., 2017a), population pharmacokinetics of temsirolimus and sirolimus in children with recurrent solid tumors: a report from the children's oncology group, genotypes were not included in the final model. Besides, in another study, developmental pharmacokinetics of sirolimus: implications for precision dosing in neonates and infants with complicated vascular anomalies, genotypes were also not included (Mizuno et al., 2017b). Most importantly, the pharmacogenomics of sirolimus is not routinely tested in clinical pediatric pharmacotherapy, and the weight-based dose recommendation in our final model is more appropriate and convenient and has better clinical practice value.

Based on a once-daily regimen or a twice-daily regimen, we simulated two initial dosing regimens. The probability of reaching the target concentrations (5–15 ng/ml) (MacKeigan and Krueger, 2015; Nathan et al., 2015) and the 95% confidence interval were both considered. For once-daily regimen, the dosages of 0.10, 0.07, 0.05, 0.04, 0.03 mg/kg/day were recommended for children with weights of 5–10, 10–20, 20–30, 30–50, and 50–60 kg, respectively; for twice-daily regimen, the dosages of 0.04, 0.03, 0.02 mg/kg/day (the daily dose was divided evenly into two doses) were recommended for children with weights of 5–20, 20–40, 40–60 kg, respectively. We found that the twice-daily regimen increased the frequency of use, but reduced the total use of sirolimus, which to some extent reduced the medical cost. Specific selection of one or two times a day for drug administration, the clinician or pharmacist can choose according to the actual clinical situation.

There were limitations in the present study, because of the low incidence of the TSC in children, the collection of patients was extremely difficult, which was also the objective reason for our small number of patients. In addition, the study is its retrospective nature and will be verified in future prospective studies.

## Conclusion

We established a population pharmacokinetic model of sirolimus in pediatric patients with TSC and the initial dosages of sirolimus in children with TSC were recommended for the first time. Large-scale pediatric TSC population needs to be validated.

## Data Availability Statement

The datasets generated for this study are available on request to the corresponding authors.

## Author Contributions

Z-PL and HX conceived and designed the study. D-DW, and XC collected the data. D-DW and XC built the model and evaluated the data. D-DW wrote the manuscript. XC reviewed and edited the manuscript. All authors contributed to the article and approved the submitted version.

## Funding

This work was supported by Clinical Pharmacy Key Specialty Construction Project of Shanghai (No. YZ2017/5). Important Weak Subject Construction Project of Shanghai (No. 2016ZB0305). Scientific research project of Science and Technology Commission of Shanghai Municipality (No. 18DZ1910604). The China Scholarship Council (No. 201906100164).

## Conflict of Interest

The authors declare that the research was conducted in the absence of any commercial or financial relationships that could be construed as a potential conflict of interest.
